# Iron Transport from Ferrous Bisglycinate and Ferrous Sulfate in DMT1-Knockout Human Intestinal Caco-2 Cells

**DOI:** 10.3390/nu11030485

**Published:** 2019-02-26

**Authors:** Xiaonan Yu, Lingjun Chen, Haoxuan Ding, Yang Zhao, Jie Feng

**Affiliations:** Key Laboratory of Animal Nutrition & Feed Science, Zhejiang Province, College of Animal Sciences, Zhejiang University, Hangzhou 310058, China; 21617026@zju.edu.cn (X.Y.); 21717018@zju.edu.cn (L.C.); 11717014@zju.edu.cn (H.D.); 21817006@zju.edu.cn (Y.Z.)

**Keywords:** DMT1, knockout, ferrous bisglycinate, ferrous sulfate, transport, intestinal

## Abstract

This experiment was conducted to investigate the transport characteristics of iron from ferrous bisglycinate (Fe-Gly) in intestinal cells. The divalent metal transporter 1 (DMT1)-knockout Caco-2 cell line was developed by Crispr-Cas9, and then the cells were treated with ferrous sulfate (FeSO_4_) or Fe-Gly to observe the labile iron pool and determine their iron transport. The results showed that the intracellular labile iron increased significantly with Fe-Gly or FeSO_4_ treatment, and this phenomenon was evident over a wide range of time and iron concentrations in the wild-type cells, whereas in the knockout cells it increased only after processing with high concentrations of iron for a long time (*p* < 0.05). DMT1-knockout suppressed the synthesis of ferritin and inhibited the response of iron regulatory protein 1 (IRP-1) and IRP-2 to these two iron sources. The expression of peptide transporter 1 (PepT1) was not altered by knockout or iron treatment. Interestingly, the expression of zinc-regulated transporter (ZRT) and iron-regulated transporter (IRT)-like protein 14 (Zip14) was elevated significantly by knockout and iron treatment in wild-type cells (*p* < 0.05). These results indicated that iron from Fe-Gly was probably mainly transported into enterocytes via DMT1 like FeSO_4_; Zip14 may play a certain role in the intestinal iron transport.

## 1. Introduction

Iron is one of the essential trace elements and a cofactor for various enzymes. It is involved in many important physiological processes, such as oxygen transport, electron transport, tricarboxylic acid cycle, and hemoglobin and myoglobin production. Iron deficiency can induce anemia and affects cell metabolism [1,2,3]. Taking into account the vital functions of iron, exogenous iron is often supplemented as food fortification and feed additive to human and animals to prevent iron deficiency [4,5,6]. However, due to the low bioavailability of inorganic iron, it often causes mineral resources waste and environmental pollution, and its abuse also affects human health and animal production [7,8,9].

The use of more efficient sources of iron is a strategy to alleviate the problems caused by excess inorganic iron. Recent studies have shown that amino acid chelated iron sources such as Fe-Gly have better iron bioavailability than inorganic iron sources such as FeSO_4_ [10,11,12,13]. The high bioavailability of amino acid chelated iron may be related to its efficient absorption and transport mechanism. However, at present, its intestinal absorption and transport mechanisms are still unclear, since there were few studies on this aspect. Some researchers thought that amino acid chelated iron might be transported across the brush border membrane (BBM) as a whole by the peptide transporter 1 (PepT1), similar to the intestinal absorption of small peptides. Their studies have shown that the intestinal uptake of amino acid chelated iron was in greater amounts and more rapid than equivalent quantities of iron salts. Furthermore, compared with FeSO_4_, Fe-Gly has been shown to significantly increase PepT1 mRNA level and protein expression in the small intestinal epithelium cells (IPEC-1) of pigs [14,15]. Others thought that amino acid chelated iron was absorbed by the enterocytes in the same manner as inorganic iron. Researchers have demonstrated that iron from the Fe-Gly competed with FeSO_4_ for the nonheme-iron absorption pathway [16]. Fe-Gly and FeSO_4_ had similar absorption kinetics characteristics and their intestinal absorptions were significantly inhibited by divalent metal ions [17,18]. In our previous study, the duodenum of Sprague–Dawley (SD) rats given Fe-Gly or FeSO_4_ were collected for transcriptome sequencing. The results showed that there was no significant difference in the expression of any iron transporter in the duodenum of rats administrated with Fe-Gly or FeSO_4_, suggesting that the amino acid chelated iron may be absorbed by the intestine in the same manner as inorganic iron, which was mainly transported into the intestinal cells via the divalent metal ion transporter DMT1 [19].

DMT1 is a key mammalian iron transporter in the enterocytes, and it is selective for the ferrous iron [20,21]. Inorganic dietary iron exists in ferric iron form predominantly; it must first be reduced by duodenal cytochrome B (DcytB) and then enters the enterocytes via DMT1 located in the apical membrane of enterocytes [22,23,24]. 

To study the iron transport from Fe-Gly in the intestinal cells, in the present study, we developed a DMT1-knockout Caco-2 cell line by using Crispr-Cas9 and treated the cells with Fe-Gly and FeSO_4_ to measure the changes of labile iron pool, ferritin content, and expression of iron regulators and transporters.

## 2. Materials and Methods 

### 2.1. Cell Culture

Human Caco-2 cells (HTB-37) were obtained from American Type Culture Collection. Cells were cultured in 25-cm^2^ flasks in Dulbecco’s Modified Eagle Medium (DMEM) (Gibco, NYC, USA) with 10% fetal bovine serum (Gibco, NYC, USA), 100 U/mL penicillin-streptomycin (Gibco, NYC, USA) and 1% non-essential amino acids (Gibco, NYC, USA) and were incubated at 37 °C, 5% CO_2_. Monolayers were grown by seeding Caco-2 cells (passage 20–40) at a density of 1 × 10^4^ cells/cm^2^ in 6-well plates or 96-well plates for about 14 days, with media changed every 2 days.

### 2.2. Knockout of DMT1 in Caco-2 Cells by Using Crispr Cas9

To generate CRISPR knockout cells, Caco-2 cells were transfected with plasmids expressing Cas9, Puro, Amp, and sgRNA for DMT1 and grown for 72 h. The sgRNAs used included 5′-AGAGAGGGATTACTATAGGCAGG-3′, 5′-CATGGGGAGTCTGCCAGTCTTGG-3′, and 5′-GAAGATCTCCATTCCTGAGGAGG-3′, which were designed based on the first exon of the public coding sequence (CDS) region located in all transcripts of DMT1. After that, the cells were screened by puromycin and separated as single cells into 96-well plates. After 2 weeks, the colonies were transferred to 48-well plates for expansion. After the cells were covered with 48-well plates, a portion (10^2^–10^4^) was removed and the Genloci TNA extraction kit (Genloci Biotechnologies Inc., Nanjing, China) was used to extract the cell genome. The following reaction system was prepared in a sterile Polymerase Chain Reaction (PCR) tube to amplify wild-type and mutant-type sufficiently hybridized DNA products. A pair of highly specific primers were designed near the knockout target site and the amplified product was approximately 316 bp in length. Primer sequences were as follows: 5′-GTATACTAAGGATGAATTGT-3′, 3′-AACCTGAGGCTGCTGAACTT-5′. The procedure used in the polymerase chain reaction (PCR) program was an initial denaturation (1.5 min at 95 °C), a three-step amplification program (10 s at 95 °C, 10 s at 62 °C, and 20 s at 72 °C) that was repeated 40 times, a complete extension (5 min at 95 °C) and a final denaturation (3 min at 72 °C). The amplified products were initially screened by the Cruiser^TM^ Enzyme (Genloci Biotechnologies Inc., Nanjing, China) to obtain positive clones, and then the clones were further verified by sequencing and western blot analysis. 

### 2.3. Measurement of Cell Viability

Wild-type and DMT1 knockout Caco-2 cells were seeded at a density of 1 × 10^4^ cells/cm^2^ in 96-well plates. After 14 days, the medium was discarded and the cells were washed twice with PBS and then incubated with DMEM containing 100 μM deferoxamine (DFO) (Sigma, St. Louis, MO, USA). Twenty-four hours later, the medium was discarded and the cells were rinsed twice with PBS before being treated with DMEM containing different concentrations of Fe-Gly and FeSO_4_ (0, 25, 50, 100, 200 μM). Two hours later, the medium was discarded and the cell viability was assessed by Cell Counting Kit-8 (MedChem Express, Princeton, NJ, USA). 

### 2.4. Measurement of Labile Iron with Phen Green SK in Wild-Type and DMT1 Knockout Caco-2 Cells

Wild-type and DMT1 knockout Caco-2 cells were seeded at a density of 1 × 10^4^ cells/cm^2^ in 96-well plates. After 14 days, the medium was discarded and the cells were washed twice with PBS and then incubated with DMEM containing 100 μM DFO. Twenty-four hours later, the medium was discarded, and the cells were rinsed twice with PBS before being treated with DMEM containing different concentrations (0, 25, 50, 100, 200 μM) of Fe-Gly (Dibo Biotechnology Co., Ltd., Shanghai, China) and FeSO_4_ (Sigma, St. Louis, MO, USA). The cells were incubated for 0, 0.5, 1, 2 h and then rinsed twice with PBS and stained with PBS containing 10 μM Phen Green SK (Thermo Fisher P14313) for 1 h at 37 °C. After staining, cells were rinsed with PBS twice and labile iron was measured by using a fluorescence microplate reader (SpectraMax M5, Molecular Devices, Sunnyvale, CA, USA) at an excitation wavelength of 490 nm and an emission wavelength of 520 nm.

### 2.5. Live Fluorescence Imaging of Labile Iron in Wild-Type and DMT1 Knockout Caco-2 Cells

To visualize labile iron, microscopy imaging of the fluorescent dye Phen Green SK (Invitrogen, Eugene, OR, USA) was performed. Wild-type and DMT1 knockout Caco-2 cells were seeded at a density of 1 × 10^4^ cells/cm^2^ in 6-well plates. After 14 days, the medium was discarded and the cells were washed twice with PBS and then incubated with DMEM containing 100 μM DFO. Twenty-four hours later, the medium was discarded and the cells were rinsed twice with PBS before being treated with DMEM containing different concentrations of Fe-Gly and FeSO_4_ (0, 25, 50, 100, 200 μM). The cells were incubated for an additional 2 h, then rinsed twice with PBS and stained with PBS containing 10 μM Phen Green SK for 1 h at 37 °C. Next, the stained cells were rinsed with PBS and observed with a fluorescence microscope (IX7, Olympus Corporation, Tokyo, Japan).

### 2.6. Western Blot Analysis 

After wild-type and DMT1 knockout Caco-2 cells in the 6-well plates were pretreated with DFO as described above, the cells were rinsed twice with PBS and then treated with DMEM containing 0 or 25 μM Fe-Gly or FeSO_4_ for 2 h. Then cells were lysed with ice-cold radio immunoprecipitation assay (RIPA) buffer (Beyotime Biotechnology, Shanghai, China) containing protease inhibitors and phenylmethanesulfonyl fluoride (PMSF, Beyotime Biotechnology, Shanghai, China) for about 1 min on ice. The lysates were transferred to a 1.5-mL centrifuge tube and were centrifuged at 11,000 × *g* for 10 min at 4 °C, then the supernatants were collected to determine the total protein concentrations using a BCA Protein Assay kit (Keygen biotech. Co. Ltd., Nanjing, China). Next, 5X dual color protein loading buffer (FD bioscience, Hangzhou, China) was added to the supernatant and then the samples were boiled for protein extraction. The extracted proteins (20–40 μg) were separated by electrophoresis on a 10% SDS-PAGE gel and transferred onto an activated polyvinylidene fluoride (PVDF) membrane (GE Healthcare Life science, Germany). Subsequently, the membrane was blocked in 5% non-fat milk at room temperature for 1 or 2 h and then incubated overnight at 4 °C with the following primary antibodies and dilution rates: DMT1, 1:500 (Santa Cruz Biotechnology, code sc-166884, Santa Cruz, CA, USA); Ferritin, 1:1000 (Abcam, code ab75973, Cambridge, UK); iron regulatory protein 1 (IRP-1), 1:1000 (Abcam, code ab126595, Cambridge, UK); IRP-2, 1:400 (Proteintech Group, code23829-1-AP, Chicago, IL, USA); hypoxia-induced factor-2α (HIF-2α), 1:1000 (Abcam, code ab207607, Cambridge, UK); PepT1, 1:200 (Abcam, code ab123314, Cambridge, UK); ferroportin 1 (FPN1), 1:2000 (Proteintech Group, code 26601-1-AP, Chicago, IL, USA); iron-regulated transporter (IRT)-like protein 14 (Zip14), 1:500 (Abcam, code ab106568, Cambridge, UK); and β-Actin, 1:2000 (Bioker biotechnology, code BK-7018, Hangzhou, China). Then the membrane was rinsed for 10 min three times thoroughly with TBST before incubation with secondary antibody consisting of goat anti-rabbit (1:20,000, Bioler biotechnology, code BK-R050) and goat anti-mouse (1:20,000, Bioker biotechnology, code BK-M050, Hangzhou, China) at room temperature for about 2 h. After that, the membrane was thoroughly rinsed with TBST for 10 min three times. The signals were detected after the addition of ECL Star Chemiluminescence solution according to the manufacturer’s instructions (Beyotime Biotechnology, Shanghai, China). 

### 2.7. Statistical Analysis 

All data are presented as the means or weighted means ± SEM of a minimum of three biological replicates unless otherwise noted. Means between groups were compared by one-way analysis of variance and post-hoc Tukey test or non-parameter Kruskal-Wallis test (SPSS software, version 21, SPSS Inc., Chicago, IL, USA) where appropriate. For this study, *p* < 0.05 was considered significant. 

## 3. Results

### 3.1. Knockout of DMT1 in Caco-2 Cells by Using Crispr Cas9

**Figure 1:**
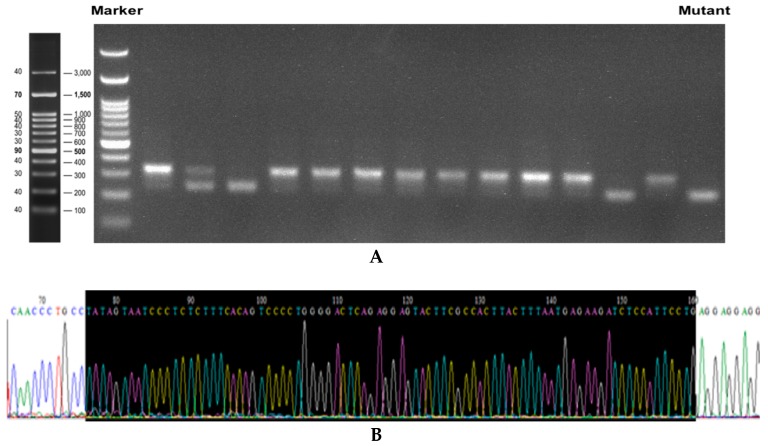

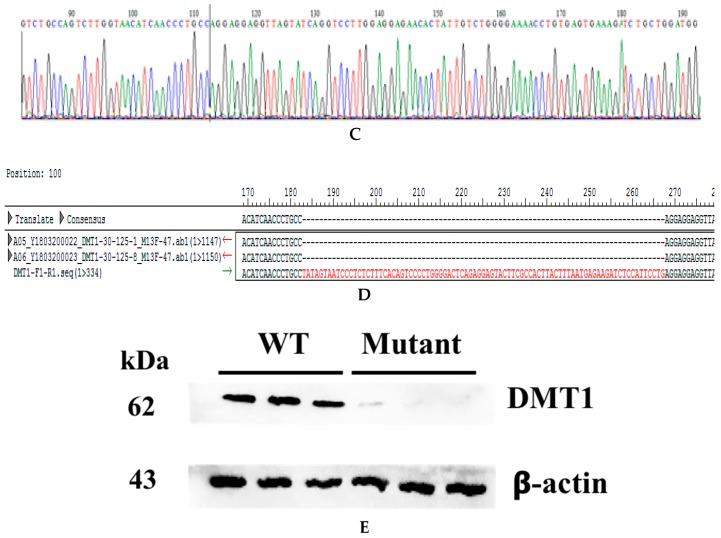
Validation of DMT1-knockout Caco-2 cell line. (**A**) The electrophoresis results of the target fragments of DMT1 in the transfected cells; (**B**) Partial sequencing results of the target fragment on DMT1 of wild-type Caco-2 cells; (**C**) Partial sequencing results of the target fragment on DMT1 of the mutant cells; (**D**) Sequence comparison of the target fragment of DMT1 in the mutant and wild-type Caco-2 cells; (**E**) Western blot results of DMT1 in wild-type Caco-2 cells and the mutant cells.

To verify the targeted disruption of DMT1 in Caco-2 cells by the Crispr-Cas9 system, we analyzed genomic DNA isolated from transfected cells using Cruiser^TM^ Enzyme assay. A 316-base pair (bp) sequence flanking the target site treated by sgRNA-encoded plasmids was amplified by PCR. As expected, the lengths of the PCR products were obviously shorter in mutant cell clones (Figure 1A). Sequencing analysis of the PCR products of these clones revealed that the mutant cells showed 85-bp deletions (5’-TATAGTAATCCCTCTCTTTCACAGTCCCCTGGGGACTCAGAGGAGTACTTCGCCACTTACTTTAATGAGAAGATCTCCATTCCTG-3’) on the exon from the DMT1 gene (Figure 1B–D). Therefore, the mutant was a positive knockout cell line on the genome. We further verified the DMT1 mutation on protein expression level. Western blot results (Figure 1E) showed that there was almost no protein expression of DMT1 in #30–125, which confirmed that the DMT1 knockout Caco-2 cell line was successfully developed.

### 3.2. Cell Viability after 2 h of Iron Treatment

**Figure 2:**
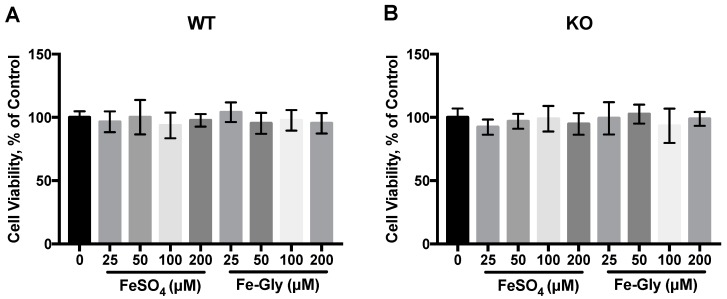
WT: wild-type Caco-2 cell; KO: DMT1-knockout Caco-2 cell. Cell viability of (**A**) wild-type and (**B**) DMT1-knockout Caco-2 cells after 2 h of iron treatment.

As shown in Figure 2, the treatment of FeSO_4_ or Fe-Gly at concentration from 25 μM to 200 μM for 2 h did not affect the viability of wild-type and DMT1 knockout Caco-2 cells.

### 3.3. Changes of Labile Iron after Treatment with Different Iron Sources

**Figure 3:**
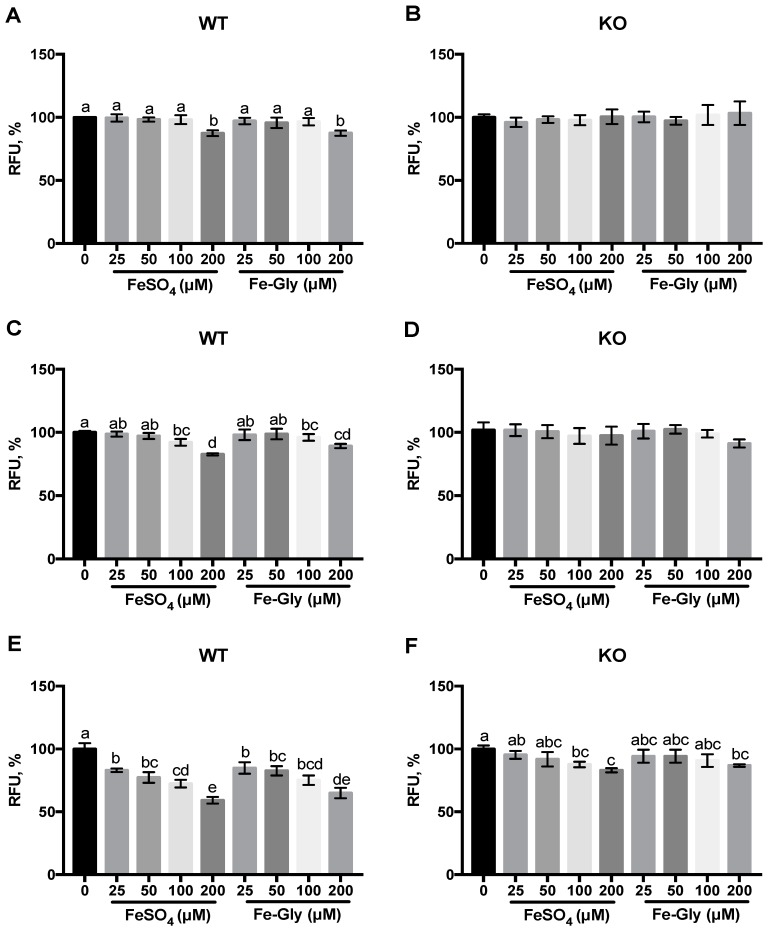
Changes of labile iron after treatment with different iron sources (0, 25, 50, 100, 200 μM) in wild-type cells and DMT1-knockout cells. Values not sharing a common letter differ significantly (*p* < 0.05). WT: wild-type Caco-2 cell; KO: DMT1-knockout Caco-2 cell; RFU: relative florescence unit. Changes of labile iron after 0.5 h (**A**,**B**), 1 h (**C**,**D**), 2 h (**E**,**F**) treatment with different iron sources in wild-type cells and DMT1-knockout cells.

The relative labile iron level was determined by using the turn-off probe Phen Green SK, which was quenched upon intracellular iron binding. After treatment with FeSO_4_ and Fe-Gly at different concentrations, the labile iron levels of wild-type Caco-2 cells and DMT1 knockout Caco-2 cells were recorded, as shown in Figure 3. 

After 30 min, 200 μM FeSO_4_ or Fe-Gly lead to significant Phen Green SK quenching in the wild-type Caco-2 cells (*p* = 0.000301, *p* = 0.000319), which indicated the significant increase of labile iron, whereas the treatment of iron had no effect on it in the knockout cells (Figure 3A,B). An hour later, the labile iron level of wild-type cells began to increase when the concentration of FeSO_4_ or Fe-Gly reached 100 μM (*p* < 0.05), while there was no change in it in the knockout cells (Figure 3C,D). Figure 3E,F show that there was a significant increase in the labile iron level in wild-type cells after treatment with 25, 50, 100, 200 μM FeSO_4_ or Fe-Gly for two hours, while that in the knockout cells elevated after processing with 100 and 200 μM iron (*p* < 0.05).

### 3.4. Live Cell Fluorescence Imaging of Labile Iron with Phen Green SK

**Figure 4:**
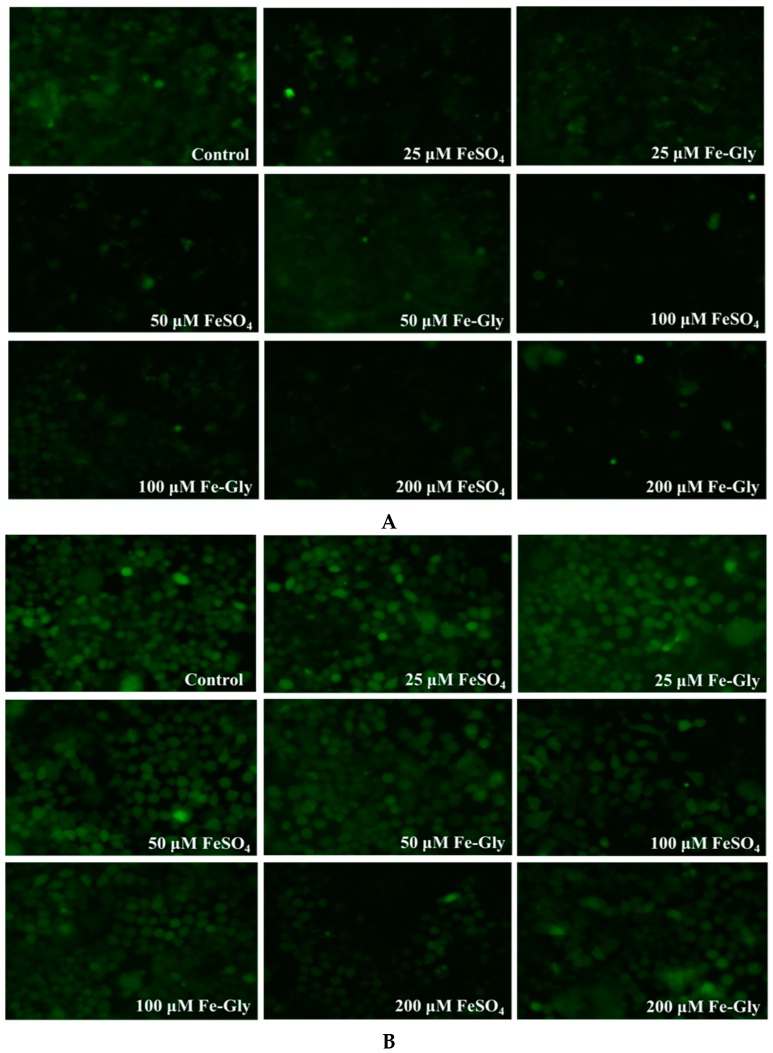
Live cell fluorescence imaging (200x) of labile iron in wild-type cells and DMT1-knockout cells after treated with different concentrations of FeSO_4_ and Fe-Gly for two hours. (**A**) Fluorescence imaging of labile iron in wild-type cells; (**B**) Fluorescence imaging of labile iron in DMT1-knockout cells.

To visualize labile iron, cells on a 6-well plate were stained with Phen Green-SK and observed by a fluorescence microscopy. The cells were treated with gradient concentrations of iron for 2 h based on the results above. It can be seen from Figure 4A,B that low concentrations of Fe-Gly or FeSO_4_ result in the quenching of Phen Green SK in the wild-type cells, which indicates the increase of labile iron, while that in the knockout cells began to change after processing with higher concentrations of iron. The results of the live cell fluorescence imaging of labile iron confirmed the results we obtained by the fluorescence microplate reader. 

### 3.5. Expression of Iron Regulators and Transporters

**Figure 5:**
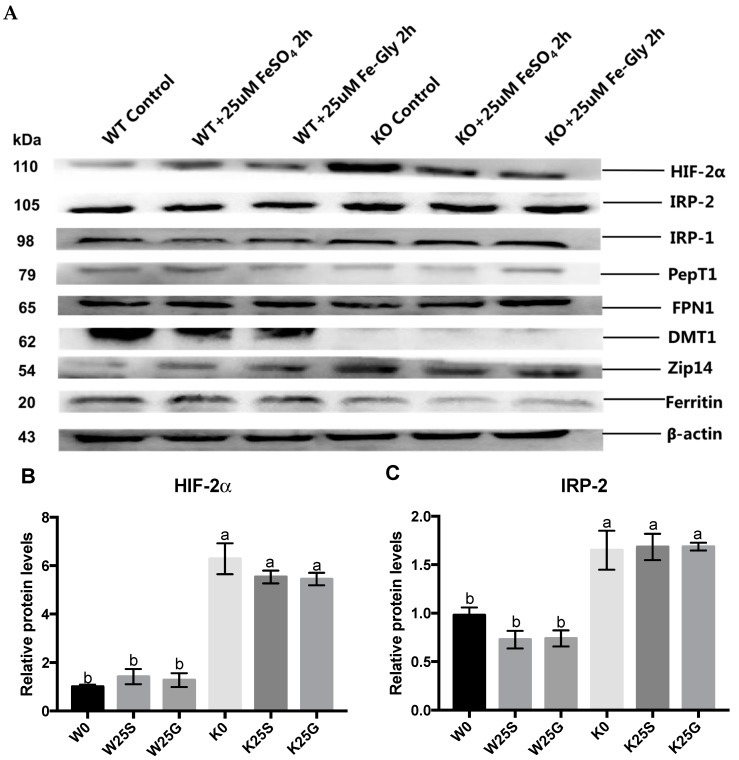

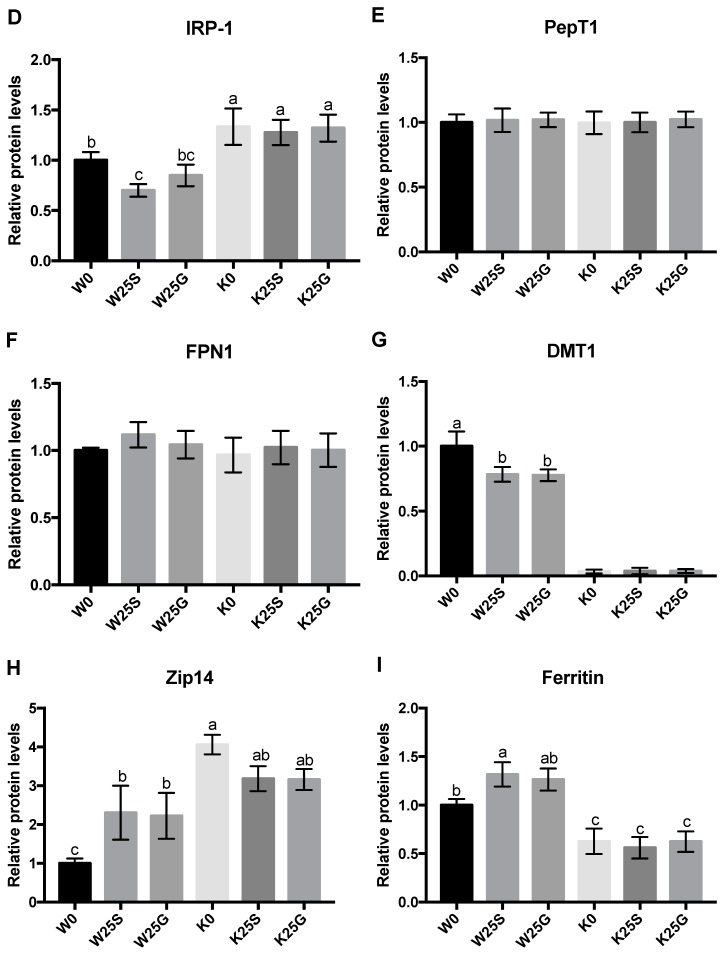
Expression of some iron regulators and transporters after treated with 25 μM of different iron sources for two hours. Values not sharing a common letter differ significantly (*p* < 0.05). W: wild-type cells treated with 0 μM iron; W25S, W25G: wild-type cells treated with 25 μM FeSO_4_, 25 μM Fe-Gly; K0: DMT1-knockout cells treated with 0 μM iron; K25S, K25G: W0: wild-type cells treated with 0 μM iron; W25S, W25G: wild-type cells treated with 25 μM FeSO_4_, 25 μM Fe-Gly; treated with 25 μM FeSO_4_, 25 μM Fe-Gly. (**A**) Western blot bands of iron regulators and transporters. Statistical analysis of (**B**) hypoxia-induced factor-2α (HIF-2α), (**C**) iron regulatory protein 1 (IRP-1), (**D**) IRP-2, (**E**) peptide transporter 1 (PepT1), (**F**) ferroportin 1 (FPN1), (**G**) DMT1, (**H**) iron-regulated transporter (IRT)-like protein 14 (Zip14), (**I**) ferritin expression.

The protein expression levels of the iron regulators and transporters were measured after cells were treated with 25 μM Fe-Gly or FeSO_4_ for 2 h and normalized to β-actin. Obviously increased expression of HIF-2α, IRP-1, and IRP-2 and decreased content of ferritin was observed after DMT1-knockout in Caco-2 cells (*p* < 0.05) (Figure 5). Furthermore, treatment of these two iron sources increased ferritin content and decreased DMT1 expression levels significantly in the wild-type cells, while those regulators in the knockout cells did not respond to the Fe-Gly or FeSO_4_ and there was almost no expression of DMT1 in the knockout cells (Figure 5). In the wild-type cells, the expression of IRP-1 was decreased significantly after treatment with FeSO_4_ (Figure 5D). Figure 5E,F show that the expression of PepT1 and FPN1 were not altered by knockout or iron treatment. Interestingly, the expression of Zip14 was elevated significantly by knockout and iron treatment in wild-type cells (*p* < 0.05) and decreased slightly after iron treatment in knockout cells (Figure 5H). 

## 4. Discussion

The transport characteristics of Fe-Gly in the enterocytes have not been clarified yet. In this study, to further investigate whether iron from Fe-Gly was transported into enterocytes like inorganic iron via DMT1, we developed a DMT1-knockout Caco-2 cell line by using Crispr-Cas9 and treated the cells with Fe-Gly and FeSO_4_ to observe the labile iron pool and determine the transport of the two forms of iron. DMT1 is the main transporter of inorganic iron in the intestine. After iron is transported into enterocytes through DMT1, it will first enter the cytosolic pool termed “labile iron pool”, which is destined for metabolism, storage, or export [23,24]. The results we obtained by using the turn-off probe Phen Green SK showed that the intracellular labile iron increased significantly with Fe-Gly or FeSO_4_ treatment, and this phenomenon was evident over a wide range of time and iron concentrations in the wild-type cells. However, in the knockout cells, the labile iron increased only after processing with high concentrations of iron for a long time (*p* < 0.05) (Figure 3 and Figure 4). These results indicated that the increase of intracellular labile iron pool after treatment with Fe-Gly or FeSO_4_ was inhibited by DMT1 knockout.

Iron from the labile iron pool that is not utilized or exported is stored in a non-toxic form within ferritin [1,2]. In this experiment, knockout of DMT1 decreased ferritin content (*p* < 0.05), and treatment with 25 μM Fe-Gly or FeSO_4_ for 2 h increased its content in the wild-type cells (*p* < 0.05) but not in the DMT1-knockout cells (Figure 5I). The results indicated that the synthesis of ferritin was suppressed by DMT1 knockout. Since ferritin synthesis reflects intracellular iron bioavailability [2,6], the greater synthesis of ferritin in the wild-type cells suggests that the iron storage and utilization from Fe-Gly were affected by the absence of DMT1 as FeSO_4_. 

Cellular iron homeostasis is mainly orchestrated post-transcriptionally by iron regulatory proteins IRP-1 and IRP-2. The two orthologous RNA-binding proteins sense the labile iron pool, and the conversion of IRP-1 to aconitine and degradation of IRP-2 will be promoted upon iron replete [25,26]. In the current study, both IRP-1 and IRP-2 expression were increased due to the absence of DMT1 (*p* < 0.05), which reflected the lower intracellular iron content in the knockout cells than the wild-type cells. The results also showed that in wild-type cells, the protein expression level of IRP-1 decreased significantly after being treating with FeSO_4_ and decreased slightly after being treated with Fe-Gly. The protein expression levels of IRP-2 decreased after treatment with both iron sources, but the difference was not significant. These results indicate the elevation of the intracellular iron levels in the cells. However, in the knockout cells, they were not altered by treatment with either of these two iron sources (Figure 5B,C). These results demonstrated that iron uptake from Fe-Gly and FeSO_4_ were suppressed by DMT1 deficiency. IRPs interact with iron regulatory elements (IREs), which are present in the 5′ or 3′ untranslated regions (UTRs) of target mRNAs. IRP-binding to IREs responds to intracellular iron levels. During iron deficiency, IRPs bind to 5′UTR IRE of mRNAs encoding genes like HIF-2α and FPN1, thus inhibiting their translation. The IRPs also appear to stimulate the translation of genes like DMT1 when bound to the 3′ UTR IRE of their mRNAs [27,28,29,30,31]. In this study, the expression of FPN1 did not change significantly by knockout or iron treatment (Figure 5F). In the current study, the cells were cultured on an ordinary well plate, which may affect the basolateral transport of iron and thus may have affected the regulation of FPN1. In addition, some isoforms of FPN1 mRNA lack IRE and thus escape the IRP regulation [2]. HIF-2α did not respond to iron treatment either, but its expression was significantly increased by DMT1 knockout (*p* < 0.05) (Figure 5B). In addition to the regulation of the IRP system, HIF-2α is directly affected by iron content, and it will be degraded after iron-mediated proline hydroxylation. Under these two different regulatory mechanisms, there was a significant increase in HIF-2α in DMT1-knockout cells [32,33,34]. It was also observed in this experiment that after treatment with the two iron sources, the expression of DMT1 was significantly decreased in wild-type cells (*p* < 0.05), while there was almost no expression in knockout cells (Figure 5G). All of the above results indicated that the iron content in the knockout cells was lower than that of the wild-type cells, and the iron absorption of the two iron sources were both affected by the knockout of DMT1. 

In addition, we also measured the expression of intestinal peptide transporter PepT1. A previous study reported that Fe-Gly significantly increases PepT1 mRNA level and protein expression in the small intestinal epithelium cells (IPEC-1) of pigs, and the reporter thought that Fe-Gly may be mediated into the intestinal cells via PepT1 [15]. However, in our study, the expression of PepT1 did not differ between wild-type and DMT1-knockout cells and between Fe-Gly and FeSO_4_ treatment (Figure 5E). This result was consistent with our previous study, which showed that there was no difference in the expression of PepT1 in the duodenum of rats administrated with Fe-Gly or FeSO_4_ [19]. 

Interestingly, when measuring the labile iron in DMT1 knockout cells, we found that the labile iron could elevate after treating with high concentrations of the two iron sources for a certain period of time. Since DMT1 was almost absent in the knockout cells and did not respond to iron treatment, we suspect that there may be other ways to mediate iron into the intestinal cells, and the transporter may be zinc-regulated transporter (ZRT) or iron-regulated transporter (IRT)-like protein 14 (Zip14). Zip14 was first proven to be a zinc transporter and recent studies have shown that it can mediate the absorption of non-transferrin bound iron (NTBI) in liver and other organs. NTBI appears the when the iron-binding capacity of plasma transferrin is exceeded under iron overload [35,36,37,38,39,40]. Recently, some studies have reported that it plays an important role in maintaining manganese homeostasis [41,42,43]. In the current study, we determined the protein expression of Zip14 and we found some interesting results. Knockout of DMT1 significantly increased the expression of Zip14 (*p* < 0.05). Furthermore, iron treatment significantly promoted Zip14 expression in wild-type cells (*p* < 0.05), while it slightly inhibited its expression in knockout cells (Figure 5H). Since DMT1 and Zip14 both can mediate the cellular uptake of metal ions, the high expression of Zip14 in knockout cells may be a complement to the lack of DMT1. Its elevation after iron treatment in wild-type cells may be due to the decreased expression of DMT1, which affected the absorption of other ions [38,39,44]. In knockout cells, its slight reduction may be in response to the iron treatment. These results supported that Zip14 may play a role in iron transport in intestinal cells. Several reports showed that the expression of Zip14 is relatively high in the intestine [38,45], but there have been few reports on its role in the intestine up until now. Here, the results of our experiments may provide some clues for further explorations and future studies.

## 5. Conclusions

The iron transport and storage from Fe-Gly in intestinal cells was affected by the knockout of DMT1 as well as FeSO_4_, which indicated that Fe-Gly was probably mainly transported into the intestine cells via DMT1 like FeSO_4_. Zip14 may play a certain role in intestinal iron transport.
